# Chemical Characterization and Wound Healing Property of *Jacaranda decurrens* Cham. (Bignoniaceae): An Experimental Study Based on Molecular Mechanisms

**DOI:** 10.1155/2020/4749712

**Published:** 2020-04-20

**Authors:** Mariana B. Serra, Wermerson A. Barroso, Cláudia Rocha, Pablo G. R. Furtado, Antônio C. R. Borges, Selma N. Silva, Marcelo M. P. Tangerina, Jessyane R. do Nascimento, Wagner Vilegas, Ademilton C. Alves, Denise F. Barbeiro, Heraldo P. de Souza, Iracelle C. Abreu, Marilene O. R. Borges

**Affiliations:** ^1^Physiological Sciences Department, Federal University of Maranhão, São Luís, Maranhão 65080-805, Brazil; ^2^Clinical Medicine Department, University of São Paulo, São Paulo 01246-903, Brazil; ^3^Department of Chemistry, Federal University of Maranhão, São Luís, Maranhão 65080-805, Brazil; ^4^DNA Medical Diagnóstics, Rio Branco, Acre 69900-622, Brazil; ^5^Institute of Biosciences, Paulista State University (UNESP), São Paulo 11330-900, Brazil; ^6^Pharmacy Department, Federal University of Maranhão, São Luís, Maranhão 65080-805, Brazil

## Abstract

**Background:**

*Jacaranda decurrens* Cham., known as carobinha, is prevalent in the Cerrado biome and presents popular use in treatment of dermatological diseases. The present study aimed to investigate the healing action of topical formulation of *Jacaranda decurrens* Cham. (FtEHJ) in mice cutaneous lesions.

**Methods:**

Phytochemical analysis of *J. decurrens* hydroalcoholic extract was carried out by using HPLC-PDA-ESI-MS and FIA-ESI-IT-MSn. Swiss mice were treated topically with formulation base (FtB) or Fibrinase® or ointment FtEHJ (15 mg/g; 50 mg/Kg). At the end of treatment periods, the inflammatory cytokines (TNF-*α*, IL-1*β,* and IL-6) in the lesions were measured by using ELISA and gene expression of TGF-*β*, Collagen I, and Collagen III was demonstrated by RTqPCR method and histological evaluation.

**Results:**

Ten compounds were identified in the extract, distributed among the classes of flavonoids and triterpenes. Treatment with FtEHJ increased the wound contraction in 24 hours, such as reduction of TNF-*α*, IL-1*β*, and IL-6 (pg/mL) cytokines in the lesion. The TGF-*β* and collagen gene expression was increased and the wound closure accelerated to nine days, with discrete inflammation, collagenization, and accented reepithelialization. Conclusions. The results obtained suggest chemical compounds present in the FtEHJ accelerates wound healing by being a gene expression modulator, and protein content of different molecules are involved in tissue repair.

## 1. Introduction

Wound healing is a physiological, multicellular phenomenon and aimed at restoring the epithelium after injury. This process depends on multiplicity of growth factors and cytokines, which contribute to regular signaling in order to alter the growth, differentiation, and metabolism of target cells. During acute wound healing, active polypeptides are present in wound bed and perform functions at all stages: inflammation, proliferation, and remodeling [1]. It has been found that disorders in wound healing are more frequent in inflammation and/or proliferation stages, which depend on interactions between different cell types and the extracellular matrix, predominantly synthesized by fibroblasts [2, 3]. Acute healing occurs along a coordinated biochemical cascade. However, a wound may become chronic if the inflammatory and proliferative phases of the cascade suffer some imbalance. Chronic wounds cause substantial morbidity, mortality, and increased health costs [4].

There are countless curative options available in the market, which are distributed from simplest coverage as solutions for hygiene and antisepsis, ointments, gels, growth factors, and even most complex types of dressings, called “smart dressings” or “bioactive dressings” [5]. However, in view of tissue repair response complexity, it is perceived that treatment with a single factor or cellular component reaches limited effectiveness in chronic wound healing. The challenge is in combined therapeutic approaches development or preferably in products development that having more than one biologically active compound, which stimulate both angiogenesis and matrix deposition and epithelial migration [6]. There has been growing increase in plants study recommended by traditional medicine due to the access ease and cultural compatibility with popular traditions, seeking to validate its use as a safe and effective herbal medicine [7, 8].

The species *Jacaranda decurrens* Cham. has a scientific synonym *Jacaranda pteroides* Silva Manso, receiving popular names such as carobinha and caroba [9]. In Brazil, it is a frequent plant in the Brazilian Cerrado of states of Goiás, Mato Grosso, Maranhão, and São Paulo [10]. The literature describes that Brazilian population uses *J. decurrens* in hypertension treatment, infectious processes, and hepatic disorders and as blood depurative [11, 12]. The infusion and decoction of leaves and stem bark have also been employed for the cutaneous healing treatment and to cure pruritus. A preparation called “garrafada” in Brazil, made from wine with roots and leaves of *J. decurrens*, is used in treatment of syphilis, rheumatism, inflammation, and dermatological diseases [9]. It has also been reported the use of teas and “garrafadas” of *J. decurrens* subsp. symmetrifoliolata for uterine and ovarian wound healing, treatment of gynecological infections, giardiasis, and amebiasis [13].

Pharmacological studies revealed that species of *Jacaranda* genus have properties such as chemopreventive, antioxidant, [14] and antimicrobial [12]. Chemical studies with *Jacaranda decurrens* revealed the presence of ursolic and oleanolic acids (triterpenes) [14]. Oleanolic acid is a metabolite that has significant antioxidant activity and ursolic acid showed antimicrobial, antiviral, hepatoprotective, immunoregulatory, and inhibitory activity of human cancer cells [15–17]. Santos et al. [18] demonstrated that the hydroalcoholic extract of *Jacaranda decurrens* leaves significantly reduced the formation of paw edema and myeloperoxidase activity in rats. Some studies on the potential efficacy of phenolic compounds in prevention or treatment of skin disorders and reduction of healing time have been published [19–21].

Although secondary metabolites with healing activity have been identified in plants of *Jacaranda* genus [14–18], there are still few studies on chemical and pharmacological activity of *Jacaranda decurrens* Cham. [11, 12, 14]. Therefore, it is necessary to study the chemical composition and cicatrizant action of it, once the population reports the use of the plant for such activity. Thus, the objectives of this work were to investigate the hydroalcoholic extract chemical composition of *J. decurrens* Cham. and evaluate the wound healing effect of a topical formulation containing the plant extract.

## 2. Materials and Methods

### 2.1. Botanical Material

The leaves of *Jacaranda decurrens* Cham. were collected in September 2013, in the municipality of São Raimundo das Mangabeiras, Maranhão, Brazil. A species sample was cataloged by Ático Seabra Herbarium of Federal University of Maranhão (UFMA) under number 1140/SLS017213.

### 2.2. Extraction and Phytochemical Analysis

The leaves were dried in air circulation in a greenhouse with a temperature of 40–45°C and blended in a DeLeo® electric mill. The powder, moderately thick (710–250 *µ*m), of the leaves was submitted to hydroalcoholic extraction by maceration with 70% ethanol in the ratio 1 : 10 (weight/volume) during 15 days, under manual shaking for 10 min once daily. The hydroalcoholic extract of *J. decurrens* (EHJ) was vacuum filtered, concentrated in a rotaevaporator (60°C), and lyophilized, obtaining a yieldof 16.19%. A part of lyophilized material was used in preparation of topical pharmaceutical formulation (FtEHJ) [22, 23].

### 2.3. LC-ESI-IT-MS/MS and FIA-ESI-IT-MSn from the Hydroalcoholic Leaves Extract of *Jacaranda decurrens* Cham

For the HPLC-ESI-IT-MS/MS and FIA-ESI-IT-MS^n^ a clean-up step was performed to remove any contaminants; the solution was purified by solid phase extraction (SPE) using Phenomenex Strata C18 cartridges (500 mg of stationary phase) that were previously activated with 5 mL of MeOH and equilibrated with 5 mL of MeOH : H_2_O (1 :  1, v/v). The compounds were eluted from cartridges using 1 mL of MeOH : H_2_O (1 : 1, v/v) with a final volume of 5 mL. The samples were then filtered through a 0.22 *µ*m PTFE filter and dried. The extract was diluted to 10 *µ*g/mL in HPLC solvent. Aliquots of 20 *µ*L were injected directly into the LC-ESI-IT-MS.

The analysis was performed on an online LC-ESI-IT-MS in a mass spectrometer LCQ Fleet, Thermo Scientific®. The analytical column used for LC separation was a Kinetex® C18 (2.1 × 100 mm, 100 Å and 5 *µ*m). The analysis was carried out with water containing 0.1% formic acid (A) and acetonitrile + 0.1% formic acid (B) added to 0.1% formic acid in exploratory gradient starting with 10% to 100% B in 6 minutes at a flow rate of 0.4 ml/min. The sample was infused into the mass spectrometer from the HPLC system, where the sample was analyzed online by ESI-MS in negative mode and UV detector associated. The mass spectra data were obtained in the same Fleet LCQ mass spectrometer from Thermo Scientific®.

For the FIA-ESI-IT-MSn assay, direct flow infusion of the samples was performed on a Thermo Scientific LTQ XL linear ion trap analyzer equipped with an electrospray ionization (ESI) source, in negative mode (Thermo, San Jose, CA, USA). A stainless-steel capillary tube at 280°C was used, with a spray voltage of 5.00 kV, capillary voltage of −90 V, tube lens of −100 V, and a 5 *µ*L min^−1^ flow. Full-scan analysis was recorded in m/z ranging from 100–1000. Multiple-stage fragmentations (ESI-MS^n^) were performed using the collision-induced dissociation (CID) method against helium for ion activation. The first event was a full-scan mass spectrum to acquire data on ions in that m/z range. The second scan event was an MS/MS experiment performed by using a data-dependent scan on the (M-H)^−^ molecules from the compounds of interest at a collision energy of 30% and an activation time of 30 ms. The product ions were then submitted to further fragmentation in the same conditions until no more fragments were observed. The identification of the different compounds in the chromatographic profile of the hydroalcoholic extract was done by comparing their retention times and UV spectra with literature data.

### 2.4. Topical Formulation Development (FtEHJ)

In preparing the FtEHJ, lyophilized extract was resuspended in propylene glycol and added to molten polyethylene glycol 4000 (PEG 4000), forming a homogeneous mixture, moss green, with pasty consistency and characteristic odor. The topical formulation final concentration was 15 mg/g. After raw materials homogenization, it was stored in containers protected from light [23].

### 2.5. Animals

Swiss mice (*Mus musculus*) were used, males aged 60 days, provided by the central animal house of the Federal University of Maranhão (UFMA). The animals were kept in the Laboratory of Research and Postgraduation in Pharmacology (LPPF) in xylan-lined polyethylene cages under light/dark cycle of 12 hours at a temperature of 22 ± 2°C and fed ration (Labina®) and water ad libitum. This work was approved by the Ethics Committee on the Use of Animals (CEUA/UFMA) under number 23115.003838/2015-58 (January 19, 2016).

### 2.6. Healing Activity

Mice (*n* = 60) were divided into the following groups: formulation base (BFt; *n* = 20); Fibrinase® (fibrinolysin 3.33 U/kg; deoxyribonuclease 2.220 U/kg; chloramphenicol 33.3 U/kg; *n* = 20) and FtEHJ (1.5 g/100 g; *n* = 20). The animals were anesthetized (8 mg/kg xylazine, 10 mg/kg ketamine). The lesions were made in the dorsal region with the aid of 1 cm diameter surgical punch. All groups received approximately 0.1 g topically from respective treatments with daily application, at the same time. During treatment the following parameters were observed: bleeding; hyperemic halo; secretion, and extension of crusts. The lesions in animals were photographed daily by digital camera coupled to a tripod at a distance of 12 cm. The lesion area analysis was done through the Image J® program, and the percentage contraction calculation was given by the following equation [24]:

(1) $$\begin{array}{c} x=\left[\left(\text{initial area}-\text{average area of the day}/\text{initial area}\right)\times 100\right]. \end{array}$$

The animals were euthanized by anesthetic overdose of xylazine and ketamine at four different times: 1st, 7th, 9th, and 12th (*n* = 5/group/treatment time). At the end of the 1st and 7th day, part of tissue was removed and stored at −20°C to perform the ELISA technique. Part of cutaneous tissue removed on the 7th day was stored with RNAlater® solution (Ambion®) at −20°C for further extraction of mRNA and tissue repair gene dosage [24, 25]. At the end of the 9th day, the removed tissue was used to make histological slides.

### 2.7. ELISA Assay

For total protein extraction, the skin tissue (Approximately 250 mg) was sprayed using a stainless steel bowl cooled for a long time in liquid nitrogen which keeps frozen samples. The powder was resuspended in 1 mL of protein lysis buffer (NP40-NP40 10%; glycerol 10%; NaCl 135 mM; Tris 20 mM; PMSF 1 mM). The samples were homogenized on a seesaw shaker for 30 minutes on ice and centrifuged (1400 rpm/30 min/4°C). The supernatant was collected, and total protein content was determined as described by Bradford, 1976 (Bio Agency®) [26]. The cytokines TNF-*α*, IL-1*β*, and IL-6 were measured in the animal's cutaneous tissue on the 1st and 7th day after treatment by quantitative ELISA analysis, using a microtiter plate reader (photometry at 505 nm), and cytokine concentrations were calculated from standard curves and are expressed as picograms per milligram of total protein, following the manufacturer's protocol R&D Systems, Minneapolis, MN, USA, based assay range: TNF-*α* 10.9–700 pg/mL; IL-1*β* 12.5–800 pg/mL; IL-6 3.1 300 pg/mL.

### 2.8. Real-Time qPCR

RNA was extracted from 250 mg of skin tissue using TRIzol® protocol (Invitrogen®). One hundred nanograms were used for real-time PCR analysis. PCR was performed in a 15 *µ*L reaction mixture containing 7.5 *µ*L 2x SYBR Green Reaction Mix (Invitrogen), 0.3 *µ*L each primer (10 pmol), 0.3 *µ*L SuperScript III RT/Platinum Taq Mix (10pmol/*µ*L), 0.15 *µ*L ROX Reference Dye, and 5 *µ*L sample in water. Gene specific primers were used. *β*2M : Sense, ACC CAC ACT GTG CCC ATC TA, Antisense, GCCACAGGATTCCATACCCA; TGF-*β* : Sense, GGA GAC GGA ATA CAG GGC TTT C, Antisense, CGG TTC ATG TCA TGG ATG GTC; Collagen I : Sense, AAA CTT TGC TTC CCA GAT GT C; Antisense, GGA CCC ATT GGA CCT GAA C; Collagen III : Sense, CCC ATG ACT GTC CCA CGT AAG C; Antisense, CCA GCT GCA CAT CAA CGA CAT C. The result was calculated under the 2^−ΔΔCT^ ratio of the housekeeping gene expression *β*2M (*β*2 microglobulin). Reactions were performed using StepOne™ Real-Time PCR System (Applied Biosystem).

### 2.9. Histology

The tissue samples removed on the 9th day after lesion treatment were embedded in 10% formaldehyde buffered solution for 24 hours, mounted in paraffin blocks, cut in 5 *µ*m sections, and stained with Hematoxylin and Eosin (HE) and Masson's Trichrome to evaluate the following parameters: inflammatory infiltrate; granulation tissue; angiogenesis; fibroblast migration; reepithelialization; and collagenization. Specimens were examined under a light microscope (100x and 400x) and scored accordingly: absent; discrete (up to 25% of the parameter); moderate (25–50%); and marked (50–100%) [25, 27].

### 2.10. Statistical Analysis

The results were expressed as mean ± standard error of the mean (SEM) using GraphPad Prism Software Inc, version 5.00 (GraphPad Software Inc., San Diego, CA, USA). Differences among groups were assessed by one-way analysis of variance (one-way ANOVA) used for multiple comparisons. A Newman–Keuls posttest was used to compare individual groups. Differences were considered statistically significant when *p* < 0.05.

## 3. Results

### 3.1. Chemical Constituents of EHJ

**Figure 1:**
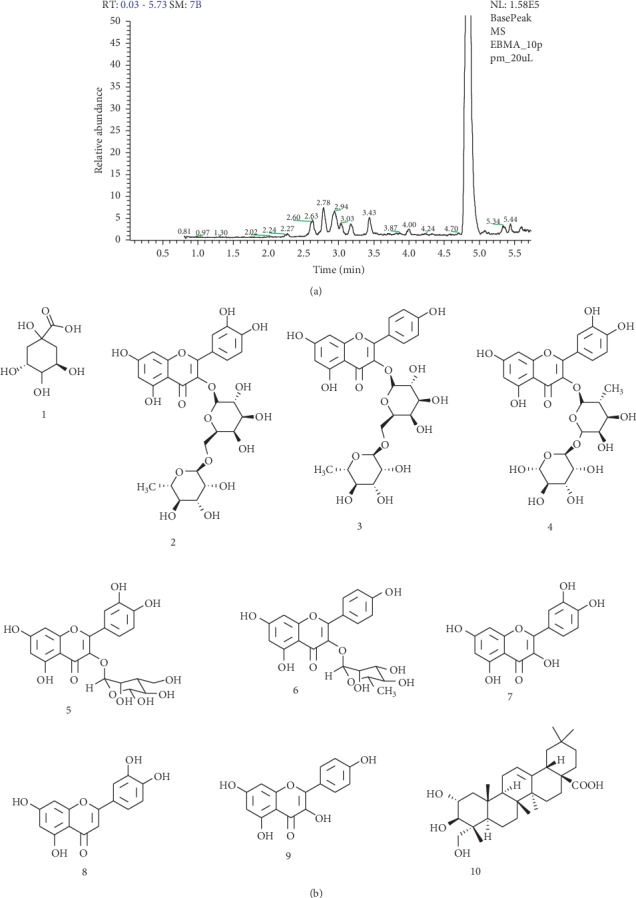
Chemical constituents of EHJ. (a) Total ion chromatogram from the hydroalcoholic extract of *Jacaranda decurrens* Cham. acquired by LC-ESI-MS (m/z: 100–1000 Da). (b) Proposed structure compounds from *Jacaranda decurrens*Cham. identified by HPLC-DAD-ESI-MS and FIA-ESI-IT-MS^n^.

The total ion chromatogram from the hydroalcoholic extract of *Jacaranda decurrens* acquired by LC-ESI-MS (m/z: 100–1000 Da) and proposed structure compounds identified by HPLC-DAD-ESI-MS and FIA-ESI-IT-MSn can be seen in Figures 1(a) and 1(b) and Table 1.

The precursor ion at m/z 191 (M-H)^−^ produced fragment ions at m/z 171, m/z 127, and m/z 85. This pattern of fragmentation led to the identification of quinic acid (1). The compound 2 showed a precursor ion at m/z 609 (M-H)^−^ in the negative ionization mode, and its MS/MS spectrum showed product ions characteristics for rutin at m/z 447 due to loss of a rhamnosyl unit, and at m/z 301, formed after loss of hexose residue (162 u) or the direct loss of rutinoside residue (rhamnosyl-glucose) unit confirmed the structure of rutin. Peak 3 showed a precursor ion at m/z 593 (M-H)^−^ and its MS/MS spectrum presented a product ion at m/z 285 attributed to the elimination of a rutinoside residue. This peak was identified as kaempferol-3-O-rutinoside.The compound 4 showed a precursor ion at m/z 595 (M-H)^−^ in the negative ionization mode, and its MS/MS spectrum showed loss of a rhamnosyl unit after loss of hexose residue (162 u) confirmed the structure of quercetin-pentoside-hexoside. Compound 5 produced a deprotonated ion at m/z 463. In analysis of the MS-MS spectrum, two high-intensity fragments at m/z 301 (M-H-162)^−^ and 300 (M-H-162)^−^ were observed. Ions at m/z 301 suggested the loss of a hexose unit. The glycosylation site of 3-OH was determined by the intensity of (M-H-162)^−^ higher than that of (M-H-162)^−^. The characteristic product ions at m/z 271, 255, 179, and 151, led to the aglycone identification as quercetin. The mass spectrum confirmed the structure quercetin-3-O-glucoside. Peak 6 showed precursor ions at m/z 431 (M-H)^−^ was identified as Kaempferol-3-O-rhamnopyranoside. The MS/MS spectrum in the negative ionization mode showed product ions at m/z 269 and m/z 353, which indicate the presence of mono-C-glycosides.

Compound 7, which produced a (M-H)^−^ at m/z 301, was tentatively established to quercetin by comparing the mass spectrum data and chromatography with an authentic standard. Peak 8 was identified as luteolin, also the MS/MS spectrum obtained in negative ionization mode presented the expected fragmentation patterns for luteolin, and the precursor ion at m/z 285 (M-H)^−^ is in agreement. In the other hand, kaempferol (peak 9) has the same molecular formula as luteolin, but the product ions formed in MS/MS spectrum in the positive ionization mode allowed distinction between them. Then, luteolin had m/z 151 as the bp, whereas kaempferol had m/z 211 as the bp and other characteristic product ion at m/z 163. Also, during the MS/MS in the negative ionization mode, luteolin showed a characteristic ion at m/z 197.Compound 10, which produced a (M-H)^−^ at m/z 487, was tentatively established to arjunolic acid by comparing the mass spectrum data and chromatography with an authentic standard.

### 3.2. The FtEHJ Accelerated Contraction of Skin Lesion in Mice

**Figure 2:**
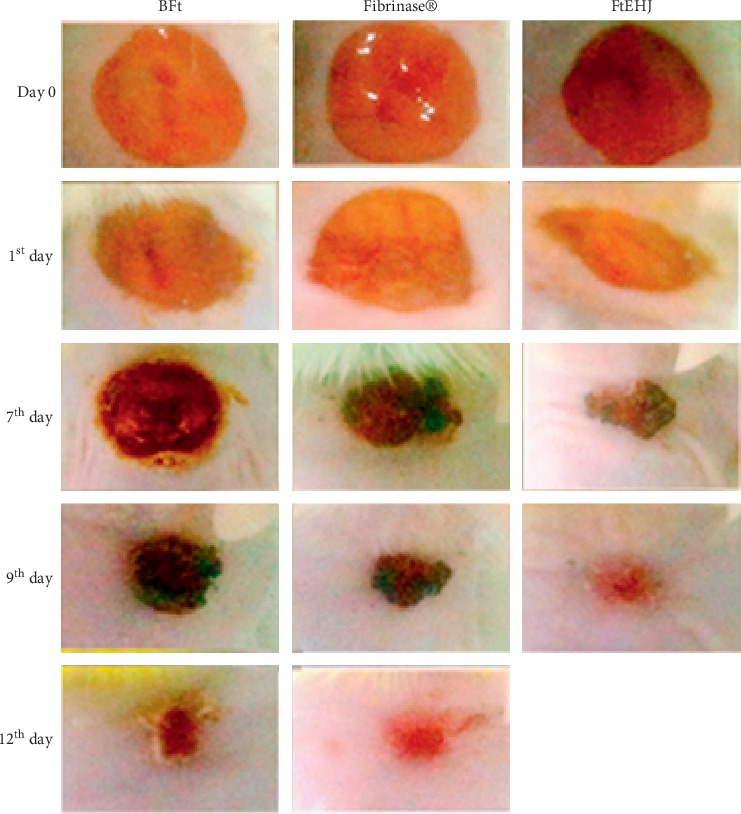
Macroscopic characteristics of the lesions. Characteristics observed in the BFt, Fibrinase®, and FtEHJ groups on the day of surgery (0 Day) and on the 1st, 7th, 9th, and 12th day after the lesion treatment. Bft, basis of the topical formulation; FtEHJ, topical formulation of the hydroalcoholic extract of *J. decurrens* Cham.

**Figure 3:**
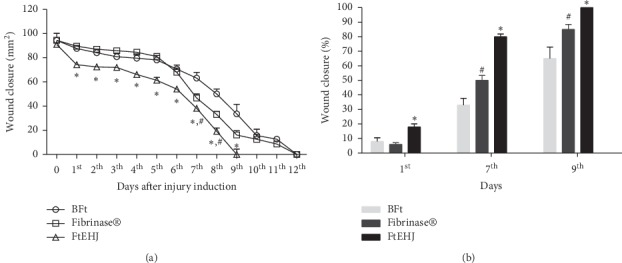
Wound closure after treatment. (a) Regression representation of wounds area (mm^2^) ± SEM of the BFt, Fibrinase®, and FtEHJ groups on the days after lesion treatment (*n* = 5/group/day after lesion treatment). (b) Percentual contraction of the lesions on 1st, 7th, and 9th day. ^*∗*^FtEHJ *vs* BFt and Fibrinase®, *p* < 0.01; ^#^Fibrinase® vs BFt, *p* < 0.01. The difference between the experimental groups was detected by analysis of variance (ANOVA), followed by Newman–Keuls posttest. Bft basis of the topical formulation; FtEHJ, topical formulation of the hydroalcoholic extract of *J. decurrens* Cham.

On the 1st day of treatment, all treated groups presented lesions with little exudative, without signs of hemorrhage or hyperemia (Figure 2). At this time, the FtEHJ group presented regression of 18% of wound total area, while in BFt and Fibrinase® groups the regressions were 8% and 6%, respectively [Figures 3(a) and 3(b)]. On the 7th treatment day, all animals showed established crusts. However, the FtEHJ and Fibrinase® groups exhibited uniform wounds and reduced crust extension when compared to BFt control (Figure 2). The FtEHJ group showed a percentage of 80% contraction, higher than BFt (33%) and Fibrinase® (50%) groups (Figure 3(b)). On the 9th day, the 5 (five) animals treated with FtEHJ had no crusts and the skin tissue was already restored (Figure 2), indicating a regression of 100% of the wound mean area, being a superior evolution when compared to BFt (65%) and Fibrinase® (85%) control groups who completely closed the lesions on the 12th day (Figures 2 and 3(b)).

### 3.3. Effect of FtEHJ on Cytokine Concentration (ELISA)

**Figure 4:**
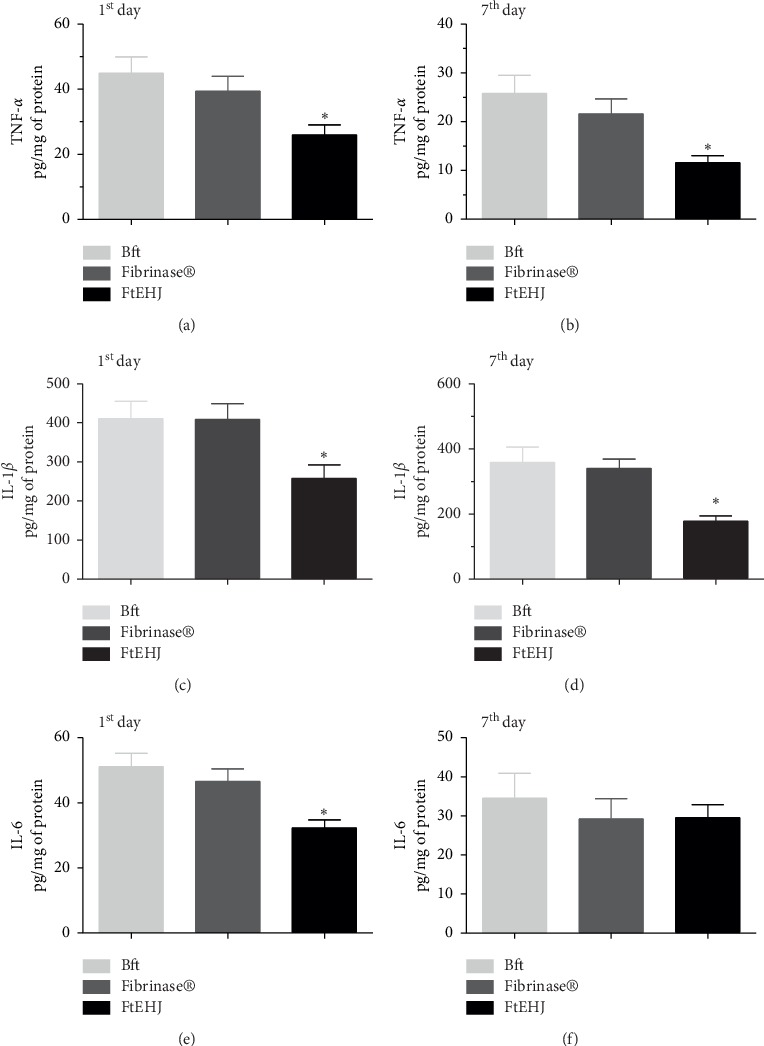
Cytokines quantification (pg/mg of protein) in cutaneous tissue after the 1st and 7th day after lesion treatment. TNF-*α* (a) 1st and (b) 7th day, IL-1*β* (c) 1st and (d) 7th day, and IL-6 (e) 1st and (f) 7th day. The results are expressed as mean ± standard error of the means (*n* = 5). The difference between the experimental groups was detected by analysis of variance (ANOVA), followed by Newman–Keuls posttest. ^*∗*^*p* < 0.01 FtEHJ *versus* BFt and Fibrinase. Bft, basis of the topical formulation; FtEHJ, topical formulation of the hydroalcoholic extract of *J. decurrens* Cham.

The proinflammatory cytokines TNF-*α*, IL-1*β*, and IL-6 dosed in the tissue after the 1st and 7th day after treatment were significantly reduced in FtEHJ when compared to the other groups (Figure 4). This reduction was also seen in IL-6, but only on the first day.

### 3.4. Effect of FtEHJ on Cytokine Gene Expression (Real-Time qPCR)

**Figure 5:**
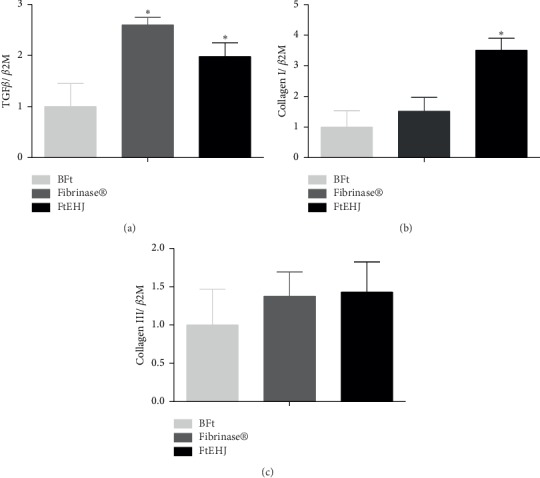
The FtEHJ increases repair genes' expression. TGF-*β* (a), collagen I (b), and Collagen III (c) in the cutaneous tissue on the 7th day after lesion treatment. The result was calculated under the 2^−ΔΔCT^ ratio of the housekeeping gene expression *β*2M and are expressed as mean ± standard error of the means (*n* = 5). The difference between the experimental groups was detected by analysis of variance (ANOVA), followed by Newman–Keuls posttest. ^*∗*^*p* < 0.01 FtEHJ or Fibrinase *versus* BFt. Bft, basis of the topical formulation; FtEHJ, topical formulation of the hydroalcoholic extract of *J. decurrens* Cham.

Figure 5 shows the effects of FtEHJ on gene expression of the molecules involved in healing process on the 7th day after injury induction. Lesions treatment with FtEHJ and Fibrinase significantly increased the gene expression of TGF-*β*. Type I collagen gene expression increased after FtEHJ treatment. There was no difference between the groups regarding the gene expression of type III collagen.

### 3.5. Histological Evaluation

**Figure 6:**
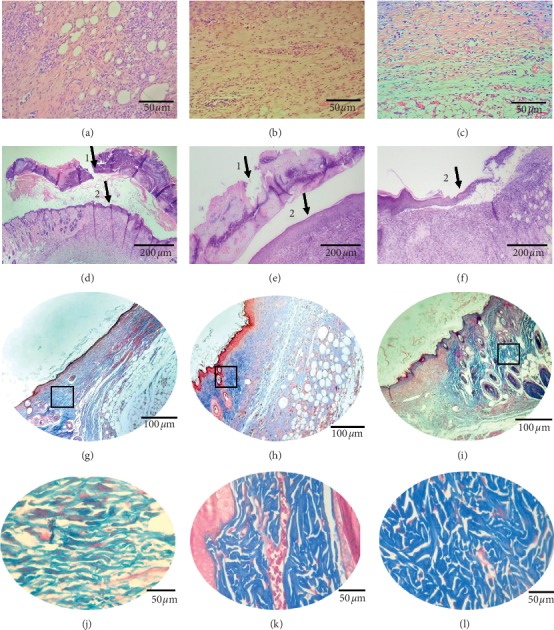
Inflammatory infiltrate and fibroblasts on the 9th day after lesion treatment of the groups. (a) BFt, (b) Fibrinase®, and (c) FtEHJ; the FtEHJ group showed less inflammation cells in tissue. (d) BFt, (e) Fibrinase®, and (f) FtEHJ;Hematoxylin and Eosin staining, using an optical microscope, 400x magnification. (1) Granulation tissue; (2) reepithelialization. The granulation tissue regressed in the FtEHJ group, while in the other groups, 100x magnification was still used. (g) BFt, (h) Fibrinase®, and (i) FtEHJ; Masson's Trichrome staining, using an optical microscope, 200x magnification. Marked area with squares is presented in higher magnification. When enlarging the images, it is noted that the (j) BFt group has collagen fibers arranged in parallel and with less intense coloring, characteristic of less dense fibers; the (k) Fibrinase® group has dense and disorganized collagen fibers, but the tissue is still moderately vascularized; and in the (l) FtEHJ group, this vascularization was already discreet, and the collagen fibers had the same characteristics found in the Fibrinase group® (400x magnification). Bft, basis of the topical formulation; FtEHJ, topical formulation of the hydroalcoholic extract of *J. decurrens* Cham.

Microscopic analysis of tissues removed on the 9th and 12th day after treatment is described in Table 2 and shown in Figure 6.

## 4. Discussion

Several studies have shown that plant extracts from polar solvents contribute to better healing, mainly by reducing inflammation, stimulating the proliferation of tissue repair cells, and collagenization [1, 3, 24, 28–30]. In this work, we observed that topical formulation of *Jacaranda decurrens* Cham. (FtEHJ) promoted a wound healing evolution homogeneously, with controlled inflammatory process and in a shorter time compared to other groups. On the first day after lesion induction, FtEHJ had promoted 18% wound regression and reduced the presence of important proinflammatory cytokines such as TNF-*α*, IL-1*β*, and IL-6. In the healing first days, these cytokines contributed to the signaling of phagocytes with the function of removing debris from the wound, but at the same time this inflammatory process can generate damage to the tissue. Modarresi et al. [1] found evidence that the topical administration of *M. piperita* ointment is able to reduce infiltration of immune cells into the wound region, and control inflammation of the tissue to promote downregulation of inflammatory cytokines and has provided acceptable efficiency in both pathological and molecular phases. Thus, interventions capable of modulating the injury inflammatory phase can be an effective strategy for wound management [31].

The chemical characterization of plant showed that the extract of *J. decurrens* Cham. presents compounds of the class of flavonoids, tannins, and triterpenes, which have been reported with anti-inflammatory, antioxidant, and healing activities [32–36]. The inflammation modulation is important to reduce the damage resulting from proteases release by neutrophils, such as excessive degradation of extracellular matrix and proteins important for tissue repair, besides the free radicals production [37]. Among these proteases the matrix metalloproteinases- (MMP-) 2 and MMP-9, which together with their inhibitors, are important for maintenance of balance between extracellular matrix degradation and collagen remodeling, and when super expressed impair tissue repair [38]. The suppressor effect of quercetin [39] and quinic acid [40] (which are also present in EHJ) on these proteases has been demonstrated. The FtEHJ modulating effect contributed to the transition process from the inflammatory phase to proliferative phase, favoring a greater wound contraction, as observed.

On the 7th day after lesion treatment, the FtEHJ already presented 80% wound retraction. This result may be associated with the lower amount of proinflammatory cytokines (TNF-*α* and IL-1*β*), characteristic of a successful proliferative phase. In contrast, IL-6 concentration was high in the group treated with FtEHJ. A study published by Zi-Qing Lin et al. [41] found that knockout mice for IL-6 showed delayed healing when compared to wild-type mice, indicating that this cytokine plays an important role in healing by leukocyte infiltration regulation, angiogenesis, and collagen accumulation. Gallucci et al. [42] observed that the delay in mice healing, after immunosuppression induction by glucocorticoids, was reversed when administered recombinant murine IL-6 (rMuIL-6). The results of this study were evidenced by the presence of epithelialization, granulation tissue formation, and wound closure. Such characteristics could be seen in the histological analysis (Figure 6), corroborating with the author's findings. It was also shown that there was greater stimulation of TGF-*β* gene expression in both the FtEHJ and Fibrinase® groups compared to the CTL group. The main component of Fibrinase® ointment is fibrinolysin, a debriding enzyme that removes fibrin and nonviable cells, facilitating the migration of fibroblasts to scar formation and tissue remodeling [43]. The increase in TGF-*β* gene expression in animals of these groups may be related to the migration and differentiation of fibroblasts in lesions, since this growth factor acts by inhibiting the matrix proteins degradation, decreasing the synthesis of metalloproteinases and increasing the production of their inhibitors as well as inducing the differentiation of fibroblasts into myofibroblasts, favoring cell migration and wound closure [44]. Different flavonoids do not play the same role in TGF-*β* expression. Kaempferol has been reported to promote suppressive activity of this molecule in a lung cancer model [45]. However, quercetin acts positively on modulation of the TGF-*β* signaling pathway, favoring healing [46]. These flavonoids are also present in propolis [47], which has healing and anti-inflammatory activity, as in other plants of *Jacaranda* genus [48, 49]. There was an increase in type I collagen gene expression in the FtEHJ group compared to the others, although type III collagen gene expression was not different between the groups. Type I collagen fiber is described in literature as more resistant to tensile strength because it has a denser characteristic than type III fiber. Therefore, the more mature the scar tissue, the greater the type I collagen fibers deposit, since during remodeling there is a renewal of collagen that changes from type III to type I [31]. The greater gene expression of collagen I in the treated group with FtEHJ may indicate more stimulus to collagen fibers organization to be produced and deposited in bigger quantity, contributing to the remodeling phase [50].

Histological evaluation made with tissues on the 9th day after lesion treatment validates the accelerating healing effects of the extract, showing characteristics of inflammatory and proliferation phases in a more discreet way and with marked collagenization and reepithelialization, indicating that the FtEHJ group was in the remodeling phase while the other groups were in the proliferative phase. At the same time, the animals treated with FtEHJ showed slight angiogenesis in the scar tissue, while in the control animals there was still moderate formation of these vessels. Since this event is related to increase of cellular metabolism, characteristic of the proliferation phase, it is possible that the animals treated with FtEHJ were already experiencing a more mature phase of healing, in transition to remodeling phase [51]. Masson's trichrome staining revealed the characterization of denser collagen fibers, organized and in greater quantity in tissues treated with FtEHJ. Collagen high levels can lead to rapid healing by increasing the tensile strength of wounds and improving the collagen fibers stabilization [52]. It was also observed that the FTEHJ group no longer presented crusts in the wounds, criterion used in the indication of experimental cicatricial process completion. The other groups remained with the crust until the 12th day after injury. These results evidenced the healing action of *Jacaranda decurrens* hydroalcoholic extract.

Considering the secondary metabolites detected in the leaf extract of this plant, the observed pharmacological effects can be attributed to the presence mainly of phenolic compounds, highlighting the flavonoids revealed in phytochemical screening and analysis by HPLC-DAD-ESI-MS and FIA-ESI-IT-MSn. This activity is mainly related to modulating effect that these compounds have on inflammatory molecules, controlling inflammation, preventing microorganism's proliferation, and contributing to rapid lesion retraction and evolution of other cicatrization stages [3, 53–55].

## 5. Conclusion

The results of this study reveal that the hydroalcoholic extract of *Jacaranda decurrens* Cham. has important phytochemical constituents of the classes of flavonoids, tannins, and triterpenes that may be involved in cutaneous wound healing. Thus, FtEHJ proved to be effective in lesions treatment by modulating the action of proinflammatory cytokines, contributing to inflammatory control, stimulating the growth factor synthesis and collagen maturation essential in cicatricial process.

## Figures and Tables

**Table 1:** Compounds identification in *Jacaranda decurrens* by LC-PDA-ESI-IT/MS and FIA-ESI-IT/MS^n^.

Peak number	Time retention (min)	UV band (nm)	(M-H)^−^	MS^n^ fragments	Compound proposed

1	2.27	299	191	171; 127; 85	Quinic acid
2	2.63	256 (max), 356	609	447; 301	Rutin
3	2.78	262 (max), 362	593	431; 285	Kaempferol-3-O-rutinoside
4	2.94	260 (max), 358	595	433; 301	Quercetin-pentoside-hexoside
5	3.06	262 (max), 356	463	301; 271; 255; 179; 151	Quercetin-3-O-glucoside
6	3.43	265 (max), 365	431	269; 253	Kaempferol-3-O-rhamnopyranoside
7	4.00	256 (max), 365	301	285; 269	Quercetin
8	4.90	254 (max), 267	285	151; 1970	Luteolin
9	5.34	262 (max), 362	285	163; 211; 227	Kaempferol
10	5.44	—	487	469; 425	Arjunolic acid

**Table 2:** Histological characteristics of cutaneous tissues at the 9th day after lesion treatment.

Characteristics	Experimental groups		
	BFt	Fibrinase®	FtEHJ
Inflammatory infiltrate	Moderate	Moderate	Discreet
Angiogenesis	Moderate	Moderate	Discreet
Fibroblastic migration	Moderate	Moderate	Discreet
Collagenization	Discreet	Moderate	Accentuated
Granulation tissue	Accentuated	Accentuated	Absent
Reepithelialization	Discreet	Accentuated	Accentuated

Score according to Rouhollahi [27]. Bft = basis of the topical formulation; FtEHJ = Topical formulation of the hydroalcoholic extract of *J. decurrens* Cham.

## Data Availability

The data used to support the findings of this study are included within the supplementary information file(s).

## Supplementary Materials

Supplementary Materials Figure S1: (A) The place where Jacaranda decurrens Cham leaves were collected. (B) Group of secondary metabolites identified in the hydroalcoholic extract of the leaves of J. decurrens Cham. (C) Jacaranda decurrens Cham. ointment promoted a fast injury closure, decreased inflammation, increased expression of tissue repair genes, and sharp collagenization.
