# Supplementary material for: Chemical Characterization and Wound Healing Property of Jacaranda decurrens Cham. (Bignoniaceae): An Experimental Study Based on Molecular Mechanisms
Source: Evid Based Complement Alternat Med. 2020 Apr 20;2020:4749712. doi: 10.1155/2020/4749712 (PMC7191437; doi:10.1155/2020/4749712)
Supplement: Supplementary Materials — Figure S1: (A) The place where Jacaranda decurrens Cham leaves were collected. (B) Group of secondary metabolites identified in the hydroalcoholic extract of the leaves of J. decurrens Cham. (C) Jacaranda decurrens Cham. ointment promoted a fast injury closure, decreased inflammation, increased expression of tissue repair genes, and sharp collagenization. [file 4749712.f1.pdf]

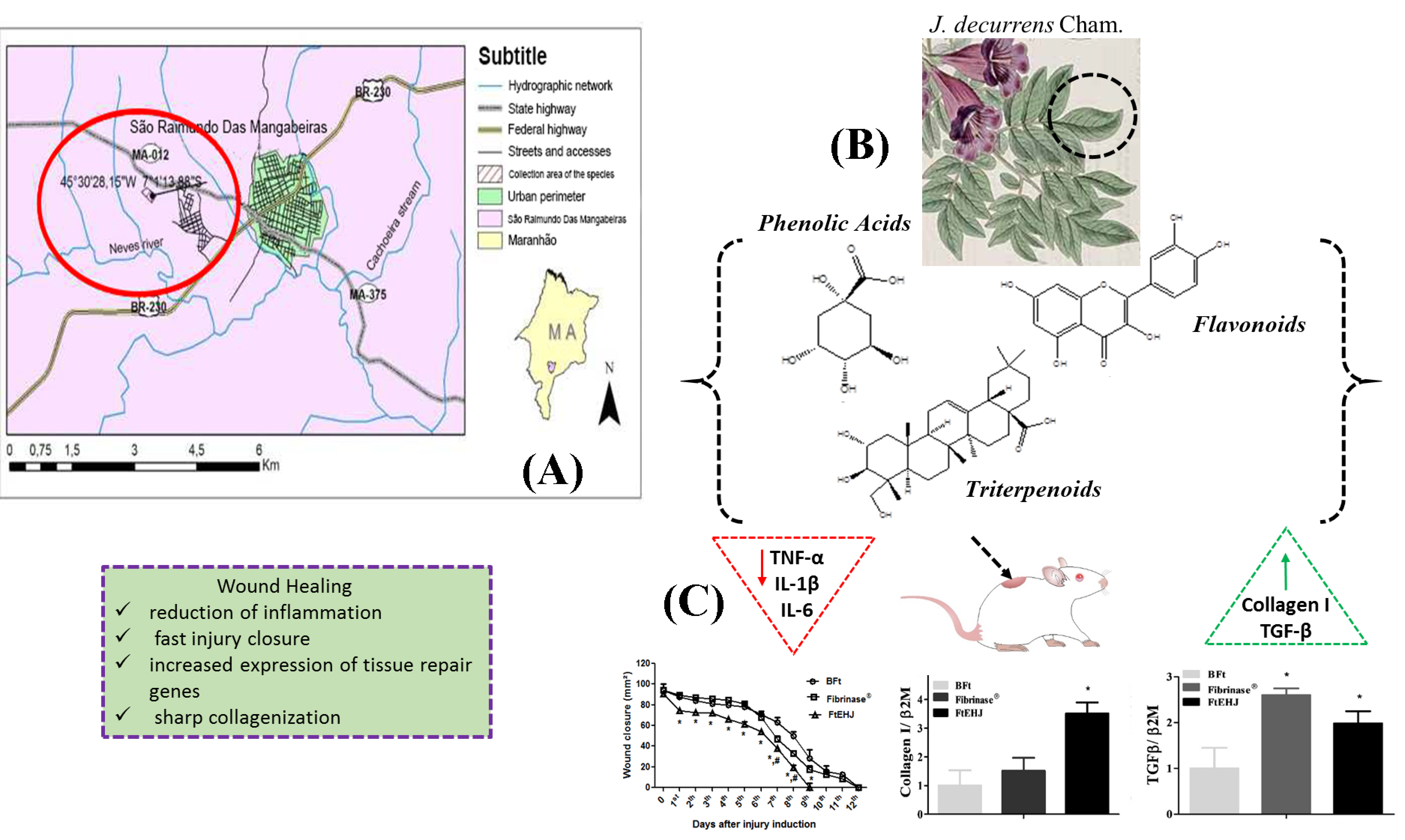
Subtitle
Hydrographic network
State highway
Federal highway
Streets and accesses
Collection area of the species
Urban perimeter
São Raimundo Das Mangabeiras
Maranhão
São Raimundo Das Mangabeiras
MA-012
45°30'28,15"W 7°1'13,88"S
Neves river
BR-230
BR-230
MA-375
Cachoeira stream
MA
N
0 0,75 1,5 3 4,5 6 Km
(A)
J. decurrens Cham.
(B)
Phenolic Acids
Flavonoids
Triterpenoids
(C)
TNF-α
IL-1β
IL-6
Collagen I
TGF-β
Wound Healing
✓ reduction of inflammation
✓ fast injury closure
✓ increased expression of tissue repair genes
✓ sharp collagenization
Wound closure (mm²)
Days after injury induction
BFt
Fibrinase®
FtEHJ
Collagen I/ β2M
TGFβ/ β2M

Figure S1: (A) Place where Jacaranda decurrens Cham leaves were collected. (B) Group of secondary metabolites identified in the hydroalcoholic extract of the leaves of J. decurrens Cham. (C) Jacaranda decurrens Cham. ointment promoted a fast injury closure, decreased inflammation, increased expression of tissue repair genes, and sharp collagenization.
